# Direct Effects, Compensation, and Recovery in Female Fathead Minnows Exposed to a Model Aromatase Inhibitor

**DOI:** 10.1289/ehp.11891

**Published:** 2008-12-12

**Authors:** Daniel L. Villeneuve, Nathaniel D. Mueller, Dalma Martinović, Elizabeth A. Makynen, Michael D. Kahl, Kathleen M. Jensen, Elizabeth J. Durhan, Jenna E. Cavallin, David Bencic, Gerald T. Ankley

**Affiliations:** 1 U.S. Environmental Protection Agency, Office of Research and Development, National Health and Environmental Effects Research Laboratory, Mid-Continent Ecology Division, Duluth, Minnesota, USA;; 2 U.S. Environmental Protection Agency, Office of Research and Development, National Exposure Research Laboratory, Ecological Exposure Research Division, Cincinnati, Ohio, USA

**Keywords:** adaptation, endocrine disruption, estradiol, fadrozole, fish, gene expression, reproduction, steroid biosynthesis, vitellogenin

## Abstract

**Background:**

Several chemicals in the environment have the potential to inhibit aromatase, an enzyme critical to estrogen synthesis.

**Objectives:**

The objective of this study was to provide a detailed characterization of molecular and biochemical responses of female fathead minnows to a model aromatase inhibitor, fadrozole (FAD).

**Methods:**

Fish were exposed via water to 0, 3, or 30 μg FAD/L for 8 days and then held in clean water for 8 days, with samples collected at four time points during each 8-day period. We quantified *ex vivo* steroid production, plasma steroids, and plasma vitellogenin (Vtg) concentrations and analyzed relative transcript abundance of 10 key regulatory genes in ovaries and 3 in pituitary tissue by real-time polymerase chain reaction.

**Results:**

*Ex vivo* 17β-estradiol (E_2_) production and plasma E_2_ and Vtg concentrations were significantly reduced after a single day of exposure to 3 μg or 30 μg FAD/L. However, plasma E_2_ concentrations recovered by the eighth day of exposure in the 3-μg/L group and within 1 day of cessation of exposure in the 30-μg/L group, indicating concentration- and time-dependent physiologic compensation and recovery. Concentration-dependent increases in transcripts coding for aromatase (A isoform), cytochrome P450 side-chain cleavage, steroidogenic acute regulatory protein, and follicle-stimulating hormone receptor all coincided with increased E_2_ production and recovery of plasma E_2_ concentrations.

**Conclusions:**

Results of this research highlight the need to consider compensation/adaptation and recovery when developing and interpreting short-term bioassays or biomarkers or when trying to predict the effects of chemical exposures based on mode of action.

In a recent report, *Toxicity Testing in the 21st Century*, the National Research Council Committee on Toxicity Testing and Assessment of Environmental Agents (2007) proposed that improved scientific understanding of toxicity pathways was central to the expanded use of predictive, pathway-based bioassays in risk assessment. Toxicity pathways can be viewed as the series of biological changes, spanning across multiple levels of biological organization, that lead from some molecular initiating event (perturbation) to an adverse outcome. A major challenge associated with dose–response modeling and extrapolation from laboratory to real-world conditions has been to understand under what conditions an organism may compensate for, or recover from, a given perturbation and under what conditions the perturbation will lead to an adverse outcome (Andersen et al. 2005). Thus, in developing useful predictive models of toxicity, we need to understand not only the direct effects of a chemical and how they translate into adverse effect, but also the potential mechanisms for compensation and recovery and how they may intersect with other biological pathways and processes.

Previous studies with the fathead minnow (*Pimephales promelas*) have suggested potential compensatory responses to the direct effects of chemicals whose primary mode of action was inhibition of one or more enzymes involved in steroid biosynthesis. For example, the chemical fadrozole (FAD) inhibits aromatase, the enzyme that catalyzes the rate-limiting conversion of testosterone (T) to 17β-estradiol (E_2_) (Miller 1988). Villeneuve et al. (2006) observed significant, concentration-dependent up-regulation of transcripts coding for the ovarian isoform of aromatase (CYP19A) in female fathead minnows exposed to FAD for 7 days. The increased *CYP19A* gene expression was associated with an inverted U-shaped concentration–response profile for ovary aromatase activity. Although that study did not examine effects on plasma E_2_ concentrations, it was noted that the responses would favor increased synthesis of E_2_ to potentially offset the effect of FAD on aromatase. In another study, Ankley et al. (2007) exposed fathead minnows to the steroidogenesis inhibitor ketoconazole for 21 days. Testosterone production by testis or ovary tissue collected from ketoconazole-exposed fish was significantly reduced compared with control fish. However, there was significant up-regulation of genes coding for cytochrome P450 cholesterol side-chain cleavage (P450scc, *CYP11A*) and cytochrome P450 c17αhydroxylase, 17,20-lyase, and concentration-dependent proliferation of steroid-producing interstitial cells in the testis of exposed males. As a result, plasma T and E_2_ concentrations in exposed fish were similar to those of controls, despite the decreased rate of steroid production per unit mass of tissue, suggesting a compensatory response to ketoconazole (Ankley et al. 2007). Both the FAD and ketocona zole studies suggest that fish have the capacity to adapt to and potentially recover from the direct effects of steroidogenesis inhibitors.

Aromatase is a key steroidogenic enzyme shown to be subject to inhibition, at least *in vitro*, by a variety of chemicals present in the environment, including certain pesticides, organochlorines, and organotins (Sanderson 2006). The aim of this study was to develop a more comprehensive understanding of molecular and biochemical responses of fathead minnows to aromatase inhibition, including direct effects, compensation, and/or recovery. We examined a time course of selected gene expression and biochemical responses over the course of an 8-day waterborne exposure to two concentrations of FAD, followed by an 8-day recovery period in control water. The experiment was designed to test several hypotheses:

- FAD would elicit direct effects consistent with its presumptive mode of action
- Over time, there would be compensatory molecular responses in females, consistent with an attempt to increase E_2_ synthesis
- Compensatory effects at the molecular level would correspond to changes in circulating E_2_ and/or rates of *ex vivo* E_2_ production
- Effects would be time and concentration dependent
- There would be recovery after cessation of FAD exposure.

Although FAD is a drug with no direct environmental relevance, its specificity makes it a useful model chemical for studying this mode of action. Results of this study provide an improved understanding of the dynamics of biological response to this chemical, and its removal. This knowledge will contribute to formulation of a robust, biologically based toxicity pathway model for the effects of estradiol synthesis inhibitors.

## Materials and Methods

### Chemical and test organisms

FAD was provided by Novartis, Inc. (Summit, NJ). All fish used in the study were reproductively mature adult fathead minnows (5–6 months of age) obtained from an onsite culture facility at the U.S. EPA Mid-Continent Ecology Division (Duluth, MN). All animals were treated humanely and with regard for alleviation of suffering, and all laboratory procedures involving animals were reviewed and approved by the Animal Care and Use Committee in accordance with Animal Welfare Act and Interagency Research Animal Committee guidelines.

### FAD exposure

We conducted exposures in 20-L glass aquaria containing 10 L ultraviolet light–treated, membrane filtered, Lake Superior water containing nominal concentrations of 0, 3.0, or 30 μg FAD/L. All treatments were delivered via water at a continuous flow of approximately 45 mL/min without the use of carrier solvents. Toxicant (and control water) delivery was initiated to 16 replicate tanks per treatment group approximately 48 hr prior to test initiation to ensure that stable FAD concentrations were achieved before adding fish. Exposures were then initiated (day 0) by transferring random groups of four male and four female fathead minnows to each tank. Fish addition times were staggered by replicate within each treatment to permit all samples from a given exposure tank to be collected within 40 min of the target exposure duration. Immediately after fish addition, each tank was furnished with four breeding substrates (poly-vinyl chloride tiles). Fathead minnows are a repeat-spawning fish species with asynchronous oocyte development that, under optimal conditions, can spawn every 3–5 days in the laboratory (Jensen et al. 2001). Males aggressively defend breeding substrates, particularly when fertilized eggs are present. Although reproduction (e.g., spawning activity, fecundity) was not considered a primary end point in this study design, supplying at least one breeding substrate per male promotes normal reproductive behaviors, provides refuge, and minimizes aggressive behavior among males.

After 24, 48, 96, and 192 hr of exposure (days 1, 2, 4, 8), we sampled fish from two replicate tanks per treatment group (a total of eight males and eight females per treatment per time point). On day 8, we terminated chemical delivery to the unsampled tanks such that all groups received FAD-free Lake Superior water for the remainder of the experiment. Sampling continued at intervals consistent with those used on days 1–8 of exposure (i.e., days 9, 10, 12, 16). Given this exposure and sampling design, days 1–8 are subsequently described as the “exposure” period of the experiment and days 9–16 are described as the “recovery” period.

During each sampling period, the fish were euthanized in a buffered solution of tricaine methanesulfonate (MS-222; Finquel; Argent, Redmond, WA), and the time at which individual fish were euthanized was recorded. We measured whole-body wet weight. Urine was expelled from males by applying gentle pressure on the fish’s abdomen and samples were collected using nonheparinized microcapillary tubes. We noted the presence or absence of sperm in the urine sample, and samples were stored at −80°C for future metabolomic analyses (e.g., Ekman et al. 2007). Blood was collected from males and females using heparinized microhematocrit tubes, and plasma was separated by centrifugation. Plasma samples were stored at −80°C until extracted and analyzed. After biofluid sampling, liver, gonads, brain, and pituitary were removed. Liver samples were transferred to preweighed microcentrifuge tubes containing a stainless steel bead (3.2 mm; Biospec Products, Bartlesville, OK) to aid later homogenization and immediately snap-frozen in liquid nitrogen. Whole gonads were weighed and divided into several pieces. The left gonad was split into two subsamples of approximately equal size, and each subsample was placed in a preweighed microcentrifuge tube containing a stainless steel bead and snap-frozen in liquid nitrogen; the anterior half was allocated for metabolomics analysis (to be reported elsewhere) and the posterior half used for RNA extraction. The posterior half of the right gonad was preserved in Davidson’s fixative for later histologic characterization, and a subsample of the anterior portion of the right gonad was used for an *ex vivo* steroid production assay. Brains were transferred to preweighed microcentrifuge tubes containing a stainless steel bead and snap-frozen in liquid nitrogen. Pituitary samples were collected with fine forceps, preserved in RNAlater (Ambion, Austin, TX) and stored at −20°C. All dissection tools were washed with RNaseZap (Ambion) between each sample to prevent cross-contamination or degradation by RNAses.

We quantified FAD concentrations in all test tanks containing fish on each sampling day of the exposure period (days 1, 2, 4, 8). Water samples collected from the tanks were analyzed by HPLC (model 1100; Agilent, Wilmington, DE). Samples (300 μL) were injected onto a 2.0 × 75 mm Synergi Hydro RP column (Phenomenex, Torrance, CA) and eluted isocratically with 57% methanol/phosphate buffer (50 mM, pH 7.0) at a flow rate of 0.25 mL/ min. We determined concentrations using a diode array absorbance at 230 nm. Tanks were also sampled on the first day of the recovery period (day 9) to determine whether FAD concentrations had diminished to nondetectable levels (method detection limit = 1 μg/L). Mean ± SD temperature, dissolved oxygen, and pH monitored during the exposure and recovery periods were 25.1 ± 0.2°C, 6.66 ± 0.55 mg/L, and 7.5 ± 0.05, respectively.

### Biochemical analyses

We conducted *ex vivo* steroid production assays using methods adapted from McMaster et al. (1995) as described previously (Ankley et al. 2007; Martinovic et al. 2008). Subsamples of gonad [15.4 ± 8.8 mg for ovary; 6.5 ± 4.0 mg for testis (mean ± SD)] were transferred to glass test tubes containing 500 μL Medium 199 (M2520; Sigma, St. Louis, MO) supplemented with 0.1 mM isobutylmethylxanthine (IBMX; Sigma I7018) and 1 μg 25-hydroxy-cholesterol (Sigma) per milliliter, on ice. At the end of each sample collection period, the tubes were transferred to a 25^o^C shaker water bath and incubated for 12 hr. After incubation, the medium from each tube was collected and stored at −20°C until analyzed, and the tissue subsample from each tube was removed and weighed. Tubes containing supplemented medium but no tissue were incubated, sampled, and analyzed along with experimental samples to serve as assay blanks.

Steroids were extracted from medium samples (*ex vivo*) or plasma samples by liquid–liquid extraction with diethyl ether and then quantified by radioimmunoassay (RIA) (Jensen et al. 2001; U.S. EPA 2002). In the case of the medium samples, E_2_ and T were measured for ovary incubations, but only T was analyzed for the testis incubations (unpublished data indicate that E_2_ production by testis samples is generally below detection limits). In general, plasma sample volumes were not sufficient to facilitate the analysis of more than one steroid; therefore, E_2_ was quantified for females and T was quantified for males. We quantified plasma concentrations of the estrogen-inducible egg yolk precursor protein vitellogenin (Vtg) by ELISA using a polyclonal antibody to fathead minnow Vtg and purified fathead minnow Vtg as a standard (U.S. EPA 2002).

### Quantitative real-time PCR analyses

We analyzed gene expression in selected ovary and pituitary samples using quantitative real-time polymerase chain reaction (QPCR). Total RNA was extracted from ovary samples using TRI Reagent (Applied Biosystems/Ambion, Austin, TX) with minor modifications to the manufacturer’s protocol. Samples were homogenized in 1.0 mL TRI Reagent using a Retsch MM300 mixer mill (Retsch Inc., Newtown, PA, USA) (30 Hz for 5 min with 3.2 mm stainless steel bead). After extraction, total RNA pellets were resuspended in RNA*secure* and DNase treated using DNA-*free* (both from Applied Biosystems/Ambion) following the manufacturer’s protocol. The RNA samples were then quantified spectrophotometrically, with absorbance at 260 and 280 nm used as a measure of relative purity and quantity. We analyzed RNA samples for structural integrity using glyoxal agarose gel electrophoresis. Total RNA was extracted from individual pituitary samples using RNeasy micro kits (Qiagen, Valencia, CA). The quality and approximate concentrations of pituitary RNA were evaluated using a Nanodrop ND-1000 spectrophotometer (Nanodrop Technologies, Wilmington, DE) and an Agilent 2100 Bioanalyzer (with an RNA Pico LabChip kit; Agilent, Palo Alto, CA). Pituitary RNA samples were diluted to approximately 2 ng total RNA/μL for use in the QPCR assays. Ovary RNA samples were diluted to 10 ng total RNA/μL. Extracted total RNA was stored at −80°C until subsequent use.

Development of QPCR assays for fathead minnow CYP19A, follicle-stimulating hormone (FSH) receptor (FSHR), CYP11A, steroidogenic acute regulatory protein (StAR), androgen receptor (AR), luteinizing hormone (LH) receptor (LHR), 20β hydroxysteroid dehydrogenase (20βHSD), LH β subunit (LH-β), and FSH β subunit (FSH-β) has been described previously (Martinovic et al. 2008; Villeneuve et al. 2006, 2007b, 2007d). Additional QPCR assays for fathead minnow transcripts coding for activin βA, progesterone membrane receptor α (PMR-α), activin receptor IIB (activin RIIB), and follistatin were developed as part of this work [see Supplemental Material (available online at http://www.ehponline.org/members/2008/11891/suppl.pdf)]. All assays were single-step real-time PCR assays conducted in a 96-well format using Taqman EZ RT-PCR kits (Applied Biosystems, Foster City, CA). Each 25-μL reaction contained either 50 ng (ovary) or 10 ng (approximate; pituitary) total RNA, 150 nM of the appropriate probe (except for FSH-β, 300 nM), 200 nM forward primer, and 200 nM reverse primer. Samples were reverse-transcribed (50°C for 2 min, 60°C for 30 min, 95°C for 5 min), followed immediately by 40 cycles of PCR amplification (melt 94°C for 20 sec, anneal and extend 58°C for 60 sec) using a 7500 Real-Time PCR system (Applied Biosystems). A standard curve of known molar quantities of gene-specific mRNA standard (10-fold dilution series, generally 50 to 5 × 10^7^ copies) was used to calibrate the QPCR data, and values interpolated from the standard curve were normalized to the mass of total RNA in the reaction and expressed as copies mRNA per nanogram total RNA for each sample. Because pituitary RNA concentrations were approximate, pituitary QPCR data were also normalized on a per-sample basis to the abundance of ribosomal protein L8 (RPL8) transcripts measured in a separate QPCR assay. Replicate (*n* = 2–4) analyses were conducted for a minimum of three samples per assay (96-well plate) to facilitate estimation of intraassay variability. Intraassay coefficients of variation (CVs) were < 10% except for the activin βA assay, which had a mean (± SD) intraassay CV of 13.2 ± 10.5% (*n* = 6 samples analyzed in triplicate). The greater variability of the activin βA assay was likely due to the fact that the low abundance (~ 500–5,000 transcripts/ng total RNA) of activin βA transcripts in ovary required greater amplification before a signal was detected [threshold cycle (Ct) values 33–36] compared with the other QPCR assays (Ct around 22–28).

### Data analysis

In general, samples from all three treatment groups (0, 3, 30 μg FAD/L, nominal) per time point were analyzed in the same assay (e.g., QPCR, RIA), whereas samples from different time points were analyzed in two to four independent assays. As a result, interassay variability did not confound statistical comparison among chemical treatments but could confound comparisons among time points. Therefore, unless otherwise stated, statistical analyses were focused on treatment comparisons within, rather than between, time points, and data are presented as fold-change (log 2) relative to the control mean for a given time point. Statistical analyses were conducted prior to conversion to fold-change (log 2) units.

We evaluated data normality and homogeneity of variance using a Kolmogorov-Smirnov test and Levene’s test, respectively. When data conformed to parametric assumptions, one-way analysis of variance (ANOVA) was used to test for differences across chemical treatment groups, and Duncan’s multiple range test was used to determine differences between chemical treatments. In cases where data did not meet parametric assumptions, data were either transformed (generally log 10) and analyzed as described above or were analyzed using a nonparametric Kruskal–Wallis test followed by Dunn’s nonparametric post hoc test. We used general linear models ANOVA to test for differences in fish mass and gonadal-somatic index (GSI) as a function of both chemical treatment, time point, and the interaction of those variables. Differences were considered significant at *p* < 0.05 unless otherwise noted. Correlations between GSI, plasma Vtg and E_2_, *ex vivo* E_2_ production, and ovarian gene expression end points (14 variables total) were examined using multivariate, correlation-based [mean centered and auto/unit variance scaled (van den Berg et al. 2006)] principal components analysis (PCA), based on data from days 1, 4, 8, 9, 12, and 16 (for six genes, QPCR data were not generated for day 2 and day 10 samples). PCA was based on a total of 136 cases, and missing data points (due to technical errors during sample collection, extraction, or analysis) were handled through mean substitution (mean for that variable across all treatments and time points). We conducted statistical analyses using SAS 9.0 (SAS Institute Inc., Cary, NC), Statistica 8 (StatSoft Inc., Tulsa, OK), and GraphPad Instat v. 3.01 (GraphPad Software, San Diego, CA).

## Results

FAD concentrations measured in water samples collected from the exposure tanks were slightly higher than the nominal target concentrations but were quite stable over the course of the 8-day exposure period. Mean (± SD) concentrations in the 3-μg FAD/L nominal treatment group were 4.2 ± 0.2, 4.0 ± 0.3, 4.1 ± 0.1, and 4.7 ± 0.1 μg/L on days 1, 2, 4, and 8, respectively. Mean (± SD) concentrations in the 30-μg FAD/L nominal treatment group were 34.6 ± 1.6, 33.6 ± 1.4, 33.0 ± 2.8, and 32.3 ± 2.6 on days 1, 2, 4, 8, respectively. No replicate tanks within a treatment deviated > 2 SDs from the mean, and no FAD was detected in water samples from control tanks. After cessation of FAD delivery, we detected no FAD in water samples collected from the exposure tanks on the first day of the recovery period (day 9). Therefore, we assumed FAD concentrations to be < 1.0 μg/L (method detection limit) for the remainder of the experiment.

FAD exposure did not significantly affect fathead minnow survival (> 99.5%) or morphologic parameters (e.g., body mass, GSI). Fish mass did not vary significantly as a function of chemical treatment group; however, males sampled on day 8 (mean ± SD, 2.99 ± 0.71 g) were significantly larger than those sampled on other days of the exposure and recovery periods (2.54 ± 0.56 g all other days) with the exception of day 12. Mean (± SD) female mass (1.25 ± 0.28 g) did not vary significantly as a function of treatment or time. Similarly, we found no significant differences in GSI among either males or females.

FAD exposure did not cause any obvious effects on fish feeding, swimming, or social behaviors. Over the course of the study (exposure and recovery phases), we noted four spawning events (207 eggs total) in the controls, four (52 eggs total) in the 3-μg/L group, and no spawns in the 30 μg/L group. However, given the design, reproduction data were not evaluated statistically.

### Steroids and Vtg

**Figure 1:**
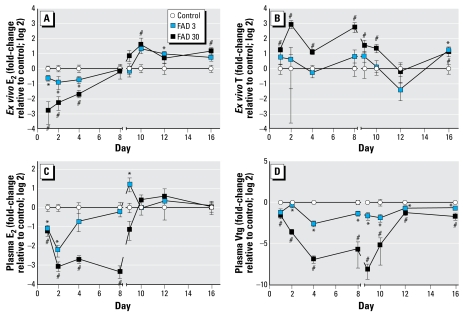
*Ex vivo* E_2_ (*A*) and T (*B*) production, and plasma E_2_ (*C*) and Vtg (*D*) measured in female fathead minnows exposed to 0 (control), 3, or 30 μg FAD/L and sampled on days 1, 2, 4, or 8 of the exposure period or on days 9, 10, 12, or 16 of the recovery period. Data are expressed as fold-change (log 2) relative to the control mean measured on a given day; error bars indicate the SE for 7–8 (*ex vivo*), 4–8 (plasma Vtg), or 3–8 (plasma E_2_) replicate fish. *p < 0.05 for 3 μg FAD/L compared with control. #*p* < 0.05 for 30 μg FAD/L compared with control.

Exposure to FAD had significant concentration- and time-dependent impacts on *ex vivo* steroid production by ovary tissue (Figure 1A, B). Twenty-four hours of exposure to either 3 or 30 μg FAD/L reduced *ex vivo* E_2_ production to 65 and 15% of control production, respectively. In the 30-μg FAD/L treatment, that effect appeared to lessen on days 2 and 4 of the exposure; by the eighth day of exposure, no significant treatment-related differences in *ex vivo* E_2_ production were detected. After cessation of FAD exposure, *ex vivo* E_2_ production by ovary tissue from the FAD-treated fish was generally greater than that by ovary tissue from controls. *Ex vivo* T production by ovary tissue was also affected by FAD treatment. Exposure to 30 μg FAD/L resulted in a significant increase in *ex vivo* T production throughout the exposure period and three of the four recovery period time points. With the exception of the T production by ovary tissues collected on day 16 (day 8 of recovery period), the lesser FAD concentration did not cause similar effects on *ex vivo* T production.

Alterations in *ex vivo* steroid production corresponded with impacts on circulating E_2_ and Vtg concentrations in female plasma (Figure 1C, D). Within 24 hr of exposure, both concentrations of FAD caused significant reductions in plasma E_2_ concentrations. Even greater depression was observed after 48 hr of exposure. However, on days 4 and 8, plasma E_2_ concentrations in fish exposed to 3 μg FAD/L recovered, whereas those in fish exposed to 30 μg/L remained depressed. After cessation of FAD exposure, plasma E_2_ concentrations rebounded to control levels within the first day of recovery in the 30-μg/L treatment group, whereas females from the 3-μg/L group had elevated E_2_ concentrations relative to the controls and then returned to control levels by the second day of the recovery period. Both concentrations of FAD depressed plasma Vtg concentrations throughout most of the exposure and recovery periods. There was a clear concentration-dependent difference in the magnitude of depression, but with the exception of day 2, the time-dependent profile of relative Vtg concentrations was largely parallel. Overall, the E_2_ and Vtg data indicate a dynamic biological response to both the presence of the chemical and its removal.

**Figure 2:**
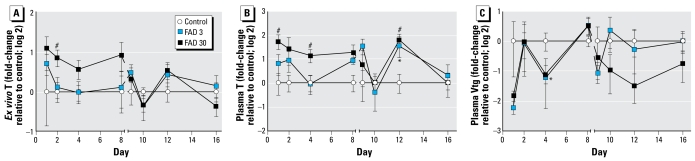
*Ex vivo* T production (*A*), plasma T (*B*), and plasma Vtg (*C*) measured in male fathead minnows exposed to 0 (control), 3, or 30 μg FAD/L and sampled on days 1, 2, 4, or 8 of the exposure period or on days 9, 10, 12, or 16 of the recovery period. Data are expressed as fold-change (log 2) relative to the control mean measured on a given day; error bars indicate the SE for 7–8 replicate fish. *p < 0.05 for 3 μg FAD/L compared with control. #*p* < 0.05 for 30 μg FAD/L compared with control.

Although FAD exposure had some significant effects on T and Vtg in males, the impacts were less substantial and trends were less clear than those observed for females. Exposure to 30 μg FAD/L appeared to increase mean *ex vivo* T production by testis tissue sampled from exposed fish; however, the effect was statistically significant on day 2 only, and production was variable during the recovery period (Figure 2A). Plasma T concentrations tended to follow a profile similar to that observed for *ex vivo* T production, and T concentrations measured in the plasma of males from the 30-μg/L treatment group sampled on days 1, 4, and 12 were significantly greater than those in control fish (Figure 2B). Vtg concentrations detected in male plasma (0.025 ± 0.27 mg/mL) were, on average, over 400-fold less than those detected in female plasma (11.1 ± 10 mg/mL). However, given the low background Vtg concentrations in males (near method detection limit), significant depression would be difficult to detect in males. Overall, we found no obvious relationship between the effects on *ex vivo* T production or plasma T and the variations in Vtg observed in males. Because of the relative lack of response of the males to the aromatase inhibitor both *ex vivo* and *in vivo*, subsequent gene expression work focused on females.

### Gene expression

**Figure 3:**
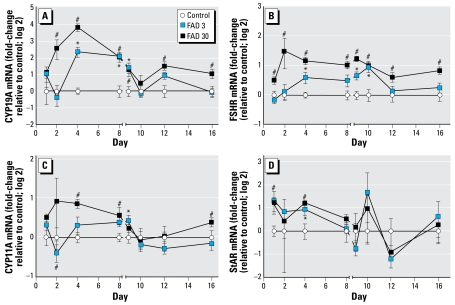
Relative abundance of mRNA transcripts coding for CYP19A (*A*), FSHR (*B*), CYP11A (*C*), and StAR (*D*) measured in ovary from female fathead minnows exposed to 0 (control), 3, or 30 μg FAD/L and sampled on days 1, 2, 4, or 8 of the exposure period or on days 9, 10, 12, or 16 of the recovery period. Data are expressed as fold-change (log 2) relative to the control mean measured on a given day; error bars indicate the SE for 5–8 replicate fish. *p **<**0.05 for 3 μg FAD/L compared with control. #*p* < 0.05 for 30 μg FAD/L compared with control.

We used real-time PCR to examine the effect of FAD exposure on the relative abundance of mRNA transcripts coding for 13 proteins thought to play important roles in regulating reproduction in female fish (Villeneuve et al. 2007c). Among the 10 transcripts examined in the ovary, 4 were significantly up-regulated at two or more time points during the exposure period (Figure 3). *CYP19A* gene expression was significantly up-regulated by day 2 in the 30-μg FAD/L treatment group and by day 4 in the 3-μg/L group. Expression remained elevated in both groups through the first day of the recovery period (day 9), then returned to control levels in the 3-μg/L group while remaining elevated in the 30-μg/L group throughout most of the recovery period (Figure 3A). Exposure to 30 μg FAD/L caused elevated *FSHR* expression throughout the entire exposure and recovery period, making it one of the most robust molecular responses to the chemical. At the lesser exposure concentration, *FSHR* induction occurred, but the effect was less rapid (significant on day 4 rather than day 1) and less persistent, with expression returning to control levels by the fourth day of the recovery period (day 12; Figure 3B). *CYP11A* and *StAR* transcripts were also induced at multiple time points. *CYP11A* was up-regulated in the 30-μg/L group on days 4 and 8 of the exposure period, as well as 8 days after cessation of FAD delivery (day 16) and in the 3-μg/L group on the first day of the recovery period (day 9). *StAR* expression was significantly greater in the ovaries of fish exposed to 30 μg FAD/L for 24 hr and in fish exposed to 3 or 30 μg FAD/L for 4 days than in control fish.

**Figure 4:**
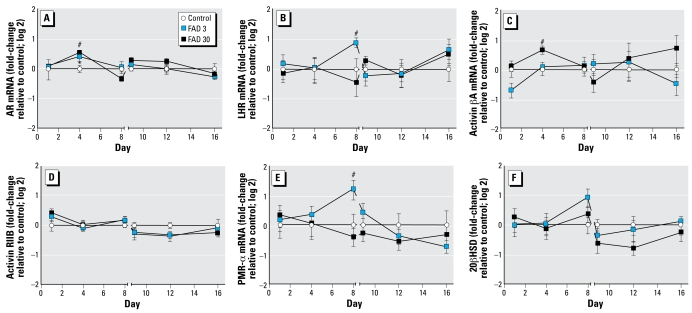
Relative abundance of mRNA transcripts coding for AR (*A*), LHR (*B*), activin βA subunit (*C*), activin RIIB (*D*), PMR-α (*E*), and 20βHSD (*F*) measured in ovary tissue from female fathead minnows exposed to 0 (control), 3, or 30 μg FAD/L and sampled on days 1, 4, or 8 of the exposure period or on days 9, 12, or 16 of the recovery period. Data are expressed as fold-change (log 2) relative to the control mean measured on a given day; error bars indicate the SE for 5–8 replicate fish. *p < 0.05 for 3 μg FAD/L compared with control. #*p* < 0.05 for 30 μg FAD/L compared with control.

Among the other six genes examined in ovary tissue, four were up-regulated, but only at a single time point (Figure 4). *AR* and activin βA were significantly up-regulated in the ovaries of fish exposed to FAD (3 and 30 μg/L for AR; 30 μg/L only for activin βA) after 4 days of exposure. In contrast, *LHR* and *PMR-*α expression was elevated after 8 days of exposure, and only in the ovaries of fish exposed to 30 μg FAD/L. Two of the genes examined, activin *RIIB* and 20β*HSD*, were not affected at any of the time points analyzed.

**Figure 5:**
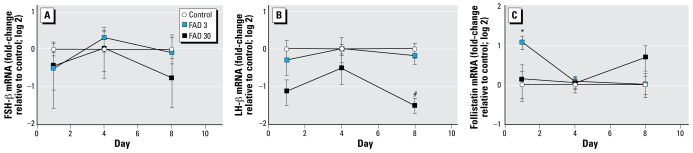
Relative abundance of mRNA transcripts coding for FSH-β (*A*), LHβ (*B*), and follistatin (*C*) measured in pituitary tissue from female fathead minnows exposed to 0 (control), 3, or 30 μg FAD/L and sampled on days 1, 4, or 8 of the exposure period. Data are expressed as fold-change (log 2) relative to the control mean measured on a given day; error bars indicate the SE for 5–8 replicate fish. *p < 0.05 for 3 μg FAD/L compared with control. #*p* < 0.05 for 30 μg FAD/L compared with control.

We examined expression of *FSH*β, *LH*β, and follistatin mRNA in the pituitary for three time points during the exposure period (days 1, 4, and 8) (Figure 5). *FSH-*β expression was unaffected by FAD exposure, but *LH*β transcripts were significantly less abundant in the pituitaries of females exposed to 30 μg FAD/L than they were in pituitaries from control fish. Follistatin expression had a very different profile: Follistatin transcripts were elevated in the pituitaries of females exposed to 3 μg FAD/L for 24 hr but were unaffected at the greater concentration and at other time points.

## Discussion

### Direct effects

The effects of FAD on *ex vivo* E_2_ production, plasma E_2_, and plasma Vtg in female fathead minnows were consistent with FAD’s anticipated mode of action. Aromatase catalyzes the conversion of C19 androgens such as T into C18 estrogens such as E_2_ (Miller 1988; Simpson et al. 1994). Consequently, we expected exposure to an aromatase inhibitor such as FAD to impair this conversion, causing a reduction in the rate of E_2_ synthesis. Consistent with this expectation, exposure to FAD for as short as 24 hr caused a concentration-dependent reduction in the concentrations of E_2_ produced by ovary tissue held in culture for 12 hr (Figure 1A). Similarly, plasma concentrations of both E_2_ and the estrogen-induced lipoprotein Vtg were significantly reduced within 24 hr of FAD exposure (Figure 1C, D). Previous studies in our laboratory have demonstrated that similar concentrations of FAD inhibit aromatase activity both *in vitro* and *in vivo* (Ankley et al. 2002; Villeneuve et al. 2006, 2007a). Consequently, we had little doubt that water-borne exposure to FAD had a rapid, direct impact on aromatase-catalyzed E_2_ synthesis.

### Compensation

Results of this study supported previous observations that fathead minnows have the capacity to mount a compensatory response to the direct, competitive inhibition of aromatase activity by increasing the transcription of *CYP19A*, presumably to increase synthesis of the enzyme (Villeneuve et al. 2006). Similar to our previous study in which concentration-dependent up-regulation of *CYP19A* gene expression was observed after 7 days of FAD exposure (Villeneuve et al. 2006), *CYP19A* was also induced in the present study. However, in the present study, this induction was time and concentration dependent, with fish exposed to 30 μg FAD/L showing significant induction earlier in the exposure than those exposed to 3 μg/L (Figure 3A). Significantly, results of this study also show a gradual recovery of *ex vivo* E_2_ production over the course of the exposure period at time points corresponding to those at which *CYP19A* gene expression was significantly elevated compared with control fish. In females exposed to 3 μg FAD/L, up-regulation of *CYP19A* expression and increases in *ex vivo* E_2_ production corresponded with the return of plasma E_2_ concentrations to levels similar to those measured in controls. As a whole, these results are consistent with the hypothesis that increased *CYP19A* expression and presumably increased translation and abundance of aromatase A serve to offset competitive aromatase inhibition by FAD and that the interplay between increased catalytic capacity and the availability (concentration) of inhibitor controls whether or not this compensation succeeds.

Results collected during the recovery period (days 9–16) provided further support for the hypothesis described above. Once the chemical was removed, *ex vivo* E_2_ production by FAD-exposed fish generally exceeded that of control fish (Figure 1A). In the case of females exposed to 3 μg FAD/L, plasma E_2_ concentrations exceeded those in control females immediately after cessation of FAD delivery, suggesting a period of overcompensation corresponding with removal of the chemical. In females from the 30-μg/L group, plasma E_2_ concentrations rebounded rapidly once FAD delivery ceased. All of these data suggest that biosynthetic capacity for E_2_ production had increased in the FAD-exposed fish and that the continued presence of the inhibitor during the exposure phase, particularly at the 30-μg/L concentration, was limiting E_2_ production.

Although *CYP19A* gene expression appears to play a key role in compensatory response to aromatase inhibition, we hypothesized that other genes involved in regulation of steroidogenesis may also be modulated. For example, both *StAR* and *CYP11A* are thought to play rate-limiting roles in steroid biosynthesis, with *StAR* regulating the rate of cholesterol transport to the inner mitochondrial membrane and *CYP11A* catalyzing the conversion of cholesterol to pregnenolone, the first step in steroidogenesis (Miller 1988; Stocco 2001; Stocco and Clark 1996). Given these roles and previous observations that expression of *CYP11A* (males and females) and *StAR* (males only) was increased in the gonads of fathead minnows exposed to keto conazole for 21 days (Ankley et al. 2007; Villeneuve et al. 2007b), we hypothesized that expression of these genes may increase in FAD-exposed females as a means to increase the overall rate of steroid biosynthesis. Results of the present study were consistent with this hypothesis. *StAR* expression was up-regulated in the ovaries of females exposed to 30 μg FAD/L for as little as 24 hr, and by the fourth day of exposure was increased in both FAD treatment groups (Figure 3D). By the eighth day of exposure and throughout the recovery period, *StAR* expression did not differ significantly from that in ovaries of control females. Statistically significant induction of *CYP11A* lagged slightly behind that of *StAR*, but by the fourth day of exposure, there was clear induction in the fish exposed to 30 μg/L (Figure 3C). *CYP11A* expression in the ovaries of females exposed to 3 μg/L peaked on the first day of the recovery period (day 9), suggesting concentration-dependence in the time course and profile of *CYP11A* expression in FAD-exposed females. Increases in mean *ex vivo* T production by ovary tissue collected from fish exposed to 30 μg FAD/L followed a time-dependent pro-file very similar to that of *CYP11A* and *StAR* expression, which suggests that up-regulation of these genes, and presumably increased translation of the corresponding proteins, may, in part, account for the increased T production we observed. However, that effect cannot be clearly discriminated from the reduced rate of converting T to E_2_ associated with aromatase inhibition. As a whole, however, *StAR* and *CYP11A* responses are consistent with a compensatory response aimed at increasing the overall rate of steroid hormone synthesis.

**Figure 6:**
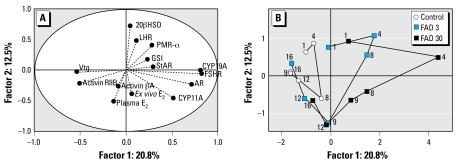
PCA of 14 variables analyzed in the ovaries of female fathead minnows exposed to 0 (control), 3, or 30 μg FAD/L and sampled on days 1, 4, and 8 of the exposure period and on days 9, 12, and 16 of the recovery period. (*A*) Loadings plot showing relative influence of each variable on variation represented by positions along factor 1 (*x*) and factor 2 (*y*). Points near the origin exert relatively little influence; those farther from the origin exert greater influence. (*B* ) Scores trajectory plot showing the mean *x* (factor 1) and *y* (factor 2) coordinates for each treatment group and time point. For clarity, *x* and *y* error bars are not shown here [the same plot with error bars is available in Supplemental Material, Figure 1 (available online at http://www.ehponline.org/members/2009/11891/suppl.pdf)].

In addition to genes coding for proteins regarded to have rate-limiting activities for steroid synthesis, we hypothesized that exposure to FAD might modulate the expression of genes associated with feedback regulation of steroidogenesis and reproductive development in the female fathead minnow. These included genes coding for *AR*, *LHR*, *FSHR*, activin βA, activin RIIB, *PMR*-α, and 20β*HSD* in the ovary and the gonadotropin β subunits and follistatin in the pituitary. By far, the most impacted of these transcripts was that coding for *FSHR*. We used PCA to examine correlations between 14 ovary-related end points and their relative contributions to treatment-related variation. Based on PCA, *FSHR* expression, along with that of *CYP19A*, had the greatest influence on variation in the positive direction along factor 1 (as indicated by relative distance from the origin along the *x*-axis) (Figure 6A). Based on PCA and on relative expression or concentration profiles for the 30-μg/L treatment group, *FSHR* expression was nearly the mirror image of plasma Vtg (Figures 1C, 3B, 6A). In contrast, the profile of *LHR* expression was most similar to that of 20β*HSD*, an enzyme key to synthesis of 17α,20β-dihydroxy-4-pregnen-3-one (17,20βP) (Nagahama 1997), and *PMR*-α, the receptor for 17,20βP (Thomas et al. 2002) (Figure 4). Together, 20β*HSD*, *LHR*, and, to a lesser extent, *PMR*-α had the greatest influence on variation in the positive direction along factor 2, whereas *ex vivo* and plasma E_2_ concentrations had the greatest negative influence on that factor (Figure 6A). AR, thought to play an important role in regulation of pre vitellogenic oocyte growth (Lokman et al. 2007; Rohr et al. 2001), also had a strong influence on variation along factor 1 and was more closely associated with the influence of *FSHR* and *CYP11A* on the PCA model than that of *LHR* expression (Figure 6A). The profiles of *FSH*-β and *LH*-β in pituitary tissue were similar to one another as a function of concentration and exposure duration (Figure 5). The associations revealed by both multi variate PCA and expression/concentration profiles for individual variables are noteworthy relative to what is known about gonadotropins and their receptors in fish.

In annual-spawning fish species such as salmonids, the gonadotropins FSH and LH appear to have functions similar to those in mammals. Specifically, FSH tends to be predominant during vitellogenesis and preovulatory growth and correlated with aromatase expression and increasing E_2_ concentrations, whereas LH production tends to be low during vitellogenesis but surges prior to ovulation, triggering increased synthesis of the maturation-inducing steroid 17,20βP, decreased aromatase expression, and final oocyte maturation (Luckenbach et al. 2008; Montserrat et al. 2004; Norris 2007; Swanson et al. 2003; Yaron 1995). However, in female repeat-spawning fish with asynchronous oocyte development such as the fathead minnow, the gonadotropins appear to be expressed in parallel throughout reproductive development (Gen et al. 2000; Kagawa et al. 2003; Kajimura et al. 2001; Villeneuve et al. 2007d; Yaron 1995), yet still coordinate the processes of vitellogenesis, follicular growth, and final maturation. Additionally, in fish, FSHR appears to bind both LH and FSH, whereas LHR appears relatively specific for LH (Bogerd et al. 2001, 2005). Given this background, the profiles of gene expression in response to FAD treatment suggest that in repeat-spawning species such as the fathead minnow, gonadotropin receptor expression in ovarian cells may be an important regulator of asynchronous oocyte development and maturation. Assuming this to be the case, increased expression of *FSHR* can be viewed as both a compensatory response to, and consequence of, impaired estrogen synthesis caused by FAD exposure.

Expression of the gonadotropins by the pituitary is regulated through negative feedback by the gonadal steroids (Yaron et al. 2001, 2003) as well as both endocrine and autocrine/paracrine effects of activin and follistatin (Cheng et al. 2007; Ge 2000; Yuen and Ge 2004). Therefore, we hypothesized that expression of activin βA, activin RIIB, or follistatin may be modulated in response to FAD exposure. Expression of activin βA, although significantly increased only on day 4, was the end point most closely associated with plasma E_2_ and *ex vivo* E_2_ production in the PCA model (Figure 6A). The gene coding for follistatin, a protein known to bind to activin, thereby suppressing *FSH*-β expression (Yuen and Ge 2004), was up-regulated only in fish exposed to 3 μg FAD/L and only after 24 hr of exposure. These results suggest at least some modulation of activin and follistatin in response to aromatase inhibition, but the overall relevance and role of that modulation relative to the other end points affected were not clear.

Reproductively mature male fathead minnows generally do not have detectable testicular aromatase activity or *ex vivo* testicular E_2_ production (Villeneuve DL, unpublished data); therefore, males were not the primary focus of this study. However, they do have some circulating E_2_ (≈ 0.4 ± 0.3 ng/mL) (Watanabe et al. 2007) and measurable brain aromatase activity that has been shown to be inhibited by FAD exposure (Ankley et al. 2002). Although the effect was not as pronounced as in females, there was a trend toward increased *ex vivo* T production and increased plasma T concentrations in males exposed to 30 μg FAD/L, which was broadly consistent with the impacts on *ex vivo* T production in females. We did not determine whether males had increases in *CYP11A* or *StAR* expression, as was observed in females.

### Recovery

Data from this experiment suggested that a repeat-spawning fish species exposed to sublethal concentrations of an aromatase inhibitor for a short time would likely recover within several days after removal of the chemical. This was indicated by the rapid recovery of plasma E_2_ concentrations to control levels after cessation of FAD delivery (Figure 1C) and a trend toward recovering Vtg concentrations. In both cases, recovery appeared to be concentration dependent, with fish exposed to 3 μg FAD/L showing some compensatory recovery while the exposure was ongoing, and those exposed to 30 μg/L recovering rapidly once FAD delivery ceased. A PCA-based scores trajectory plot also provided evidence of an overall concentration-dependent recovery. The mean scores for the control fish showed a trajectory over the course of the experiment that traced a small loop (Figure 6B). By the first day of the exposure period, mean scores for both FAD treatment groups fell outside of the control trajectory loop. The greatest deviation from the control trajectory was observed on the fourth day of exposure for both FAD treatment groups. However, after day 4, trajectories moved back in the direction of the control group. By the eighth day of the recovery period (day 16), mean scores for the 3-μg/L treatment group were effectively contained within the loop of the control trajectory. Also, mean scores for the 30-μg/L group on the fourth day of recovery (day 12) were nearly identical to those observed for the 3-μg/L group on the first day of recovery (day 9), whereas those on the eighth day of recovery (day 16), were similar to those observed on the fourth day of recovery (day 12) in the 3-μg/L group. Thus, our data support the conclusion that recovery is possible and that the time course of compensation and recovery was concentration dependent.

## Conclusions

Studies conducted with FAD and other aromatase-inhibiting chemicals have led to the formulation of a conceptual toxicity pathway model for the effects of these chemicals on fish reproduction. Specifically, the direct action of FAD inhibits aromatase activity in females, leading to significant declines in rates of E_2_ synthesis, plasma E_2_ concentrations, Vtg expression in the liver, and plasma Vtg concentrations, as well as reduced uptake of Vtg into developing oocytes, and ultimately reduced fecundity (Ankley et al. 2002; Villeneuve et al. 2006, 2008, the present study). Compensatory responses to aromatase inhibition include increased expression of transcripts coding for aromatase A, proteins thought to be rate limiting for steroidogenesis (e.g., *StAR*, *CYP11A*), and *FSHR* as a potential key regulator of vitellogenesis and preovulatory development in the ovary of repeat-spawning female fish. These compensatory responses appear capable of offsetting the direct effects of the chemical, but the time course and efficacy of compensation is dependent on the chemical concentration. Further, after the inhibitor is removed, fish appear able to recover from the effects of the perturbation, but the time course of recovery is concentration dependent and may also vary with the duration of exposure. Similar time-course experiments with other steroidogenesis inhibitors should help establish whether the observations made in this study are generalized responses to steroidogenesis inhibition or disruption of vitellogenesis, or whether they are specific to aromatase inhibition, thereby helping to define the overall applicability of this toxicity pathway model.

Additionally, the results of this study have significant implications relative to the development of short-term testing protocols or biomarkers for detecting and identifying endocrine-disrupting chemicals (or other sublethal toxicants). For example, based on *ex vivo* E_2_ production results alone, a 24-hr test protocol would have identified FAD as a likely endocrine disruptor, whereas an 8-day test protocol would not. Because of their rapid, sensitive, and persistent responses, end points such as plasma Vtg or ovarian FSHR or *CYP19A* expression would likely have greater utility as biomarkers than other end points that show more transient responses either during exposure or recovery. Development of robust toxicity pathway models that incorporate compensation and recovery mechanisms should aid in predicting toxicologic outcomes after exposure to specific classes of toxicants.
